# Evidence of Modular Responsiveness of Osteoblast-Like Cells Exposed to Hydroxyapatite-Containing Magnetic Nanostructures

**DOI:** 10.3390/biology9110357

**Published:** 2020-10-25

**Authors:** Stefania Scialla, Barbara Palazzo, Alessandro Sannino, Tiziano Verri, Francesca Gervaso, Amilcare Barca

**Affiliations:** 1 Department of Engineering for Innovation, University of Salento, 73100 Lecce, Italy; barbara.palazzo@enea.it (B.P.); alessandro.sannino@unisalento.it (A.S.); francesca.gervaso@nanotec.cnr.it (F.G.); 2 Institute of Polymers, Composites and Biomaterials—National Research Council, Viale J. F. Kennedy, 54 (Mostra d’Oltremare Pad.20), 80125 Naples, Italy; 3 ENEA, Division for Sustainable Materials—Research Centre of Brindisi, 72100 Brindisi, Italy; 4 Laboratory of Applied Physiology, Department of Biological and Environmental Sciences and Technologies (DiSTeBA), University of Salento, 73100 Lecce, Italy; tiziano.verri@unisalento.it; 5 CNR Nanotec—Institute of Nanotechnology, 73100 Lecce, Italy

**Keywords:** magnetic nanocomposites, bone cells, biomimicry, physiological responsiveness

## Abstract

**Simple Summary:**

Current research on nanocomposite materials with tailored physical–chemical properties is increasingly advancing in biomedical applications for bone regeneration. In this study, occurrence of differential responsiveness to dextran-grafted iron oxide (DM) nanoparticles and to their hybrid nano-hydroxyapatite (DM/n-HA) counterpart was investigated in human-derived, osteoblast-like cells. Sensitivity of cells in the presence of DMs or DM/n-HAs was evaluated in terms of cytoskeletal dynamics. Remarkably, it was shown that effects triggered by the DM are no more retained when DM is embedded onto DM/n-HA nanocomposites. In parallel, analyses on the expression of genes involved in (a) intracellular signaling pathways triggered by ligands or cell interactions with elements of the extracellular matrix, (b) modulation of processes such as cell cycle arrest, apoptosis, senescence, DNA repair, metabolism changes, and (c) iron homeostasis and absorption through cell membranes, indicated that the DM/n-HA-treated cells retain tracts of physiological responsiveness unlike DM-treated cells. Overall, a shielding effect by the n-HA was assumed (masking the DM’s cytotoxicity), and a modular biomimicry of the DM/n-HA nanocomposites. On these bases, the biocompatibility of n-HA associated to DM’s magnetic responsiveness offer a combination of structural/functional features of these nano-tools for bone tissue engineering, for finely acting within physiological ranges.

**Abstract:**

The development of nanocomposites with tailored physical–chemical properties, such as nanoparticles containing magnetic iron oxides for manipulating cellular events at distance, implies exciting prospects in biomedical applications for bone tissue regeneration. In this context, this study aims to emphasize the occurrence of differential responsiveness in osteoblast-like cells to different nanocomposites with diverse features: dextran-grafted iron oxide (DM) nanoparticles and their hybrid nano-hydroxyapatite (DM/n-HA) counterpart. Here, responsiveness of cells in the presence of DMs or DM/n-HAs was evaluated in terms of cytoskeletal features. We observed that effects triggered by the DM are no more retained when DM is embedded onto the DM/n-HA nanocomposites. Also, analysis of mRNA level variations of the focal adhesion kinase (*FAK*), *P53* and *SLC11A2/DMT1* human genes showed that the DM/n-HA-treated cells retain tracts of physiological responsiveness compared to the DM-treated cells. Overall, a shielding effect by the n-HA component can be assumed, masking the DM’s cytotoxic potential, also hinting a modular biomimicry of the nanocomposites respect to the physiological responses of osteoblast-like cells. In this view, the biocompatibility of n-HA together with the magnetic responsiveness of DMs represent an optimized combination of structural with functional features of the DM/n-HA nano-tools for bone tissue engineering, for finely acting within physiological ranges.

## 1. Introduction

The possibility of driving the physiological responses of living cells is one of the main challenges in the research field of nano-biomaterials applied to life science. In this view, a major part of current research is focused on the development of tools with tailored physical–chemical properties, among which there are magnetic nanoparticles (MNPs) based on iron oxides, capable of allowing the manipulation of cellular events at distance due to their sensitivity/responsiveness to magnetic fields [1]. At cellular level, activated MNPs are able to exert magnetic actuation by triggering stimuli via mechanical, thermal, or molecular perturbations; this opens new exciting scenarios and improvements to the field of drug delivery and tissue engineering applied to e.g., regenerative or anticancer medicine [1,2].

In recent years, researchers have enhanced their investigations to understand how MNPs can be manipulated at different levels (e.g., dimensional, bulk/surface physical–chemical, structural, morphological and magnetic) in order to improve the biomimicry of the biomaterials used as nano-systems for delivery, taking inspiration from the extracellular environment, from the molecular structures of cell-cell communication, and from cellular feedback from external stimuli [3,4]. In a general sense, a large category of MNPs consists of nanoparticles with a magnetic core (usually composed of magnetite Fe_3_O_4_ or maghemite γ-Fe_2_O_3_) that confers superparamagnetic features, so that they are magnetized by the application of weak magnetic fields and then demagnetize at room temperature once the field is removed. This property allows useful handling/activation of MNPs towards target cells, tissues, or organs. MNPs cores’ surface functionalization or coating with an inert layer (polymeric or inorganic), enhances MNPs’ colloidal dispersion and biocompatibility [5]. However, they do not control the MNP rapidly degradation after completing their function. To overcome this limit, MNPs encapsulation within biodegradable polymers was investigated as microenvironment for precisely controlled-degradation of MNPs in physiological conditions [6].

MNPs represent a feasible multi-purpose option for tissue engineering even if their application in bone tissue is still challenging. In fact, despite the literature describing that iron can influence bone health [7,8], to date its use in bone tissue regeneration/repair remains not systematically investigated [9,10,11]. To adopt MNPs as effective tool for bone tissue regeneration, one of the basic requirement resides in avoiding and/or controlling their toxicity features [12]. In this view, a feasible approach is the design of non-toxic MNPs by combining their superparamagnetic core phase with an inorganic/organic ‘sheltering’ counterpart, represented in this case by nano-hydroxyapatite (n-HA) [Ca_10_(PO_4_)_6_(OH)_2_] [11,12]. This approach has dual value: a) on one hand, the magnetic component provides additional effectiveness to the HA; b) on the other hand, n-HA enhances the biomimicry of Fe-based MNPs, in terms of structural biocompatibility and osteoconductivity [13]. It is already known that the surface roughness of n-HA functionalized with metal-based nanoparticles improves the growth rate of osteoblasts (bone forming cells) more than metals alone do, i.e., enhancing adhesion, proliferation and differentiation of cells, providing a faster bone regeneration [14,15,16,17]. Also, it has been confirmed by early evidence that maghemite nanoparticles coated with hydroxyapatite are more inductive to osteoblast growth than their uncoated counterpart [14,15]. Later, it has been demonstrated that human osteoblast cells show an extensive network of filopodia related to the focal adhesion points, when cells grow in the presence of iron oxide/n-HA nanocomposites [16,17]. Even if cell-material interactions are still far from being completely characterized, these results generally suggest a key potential of iron oxide/n-HA nanocomposites in actively driving bone growth site-directed in bone defects.

In this context, this study aims to emphasize the differential responsiveness of the MG63 human-derived osteoblast-like cells to different magnetic nanocomposites with diverse features: i.e., dextran-grafted iron oxide (DM) nanoparticles and their hybrid nano-hydroxyapatite (DM/n-HA) counterpart, which were structurally characterized in our previous work [18]. The MG63 cells exhibit irrelevant mineralization with respect to other cell lines, thus they are suitably useful to study cell behavior in the presence of exogenous mineralizing elements [19]. Here, the physiological responses of cells in the presence of DMs or DM/n-HAs were evaluated in terms of morphological features and gene expression variations of the *FAK* (focal adhesion kinase), *P53* (*TP53*, tumor protein 53) and *SLC11A2/DMT1* (divalent metal transporter 1) genes, taken together as a minimal networking marker in the physiological-to-pathophysiological range of cellular responses potentially triggered by different MNP compositions.

## 2. Materials and Methods

### 2.1. Chemicals and Reagents

For DM and DM/n-HA nanostructures synthesis, iron (II) chloride tetrahydrate (FeCl_2_·4H_2_O, ≥99%), iron (III) chloride hexahydrate (FeCl_3_·6H_2_O, ≥97%) and Dextran from *LeuconostocMesenteroids* (Mw 6000 Da) were purchased by Alfa Aesar. Sodium hydroxide anhydrous pellets (NaCl, ≥99%), hydrochloric acid (≥37% wt. in water), phosphoric acid (≥85% wt. in water), ammonium hydroxide ((NH_4_)OH, ≥30% wt. in water), and calcium acetate hydrate (Ca(CH_3_COO)_2_·XH_2_O, ≥99%) were purchased from Sigma–Aldrich. Ultrapure water (18.2 MΩ/cm, obtained by a Milli-Q^®^ Direct Water Purification System, Merck Millipore, Darmstadt, Germany) has been used in all the experiments.

For the cell-based experiments, Eagle’s Minimum Essential Medium (E-MEM), fetal bovine serum (FBS), L-glutammine, penicillin/streptomycin antibiotic mix, Dulbecco’s phosphate buffer saline (D-PBS), trypsin-EGTA, paraformaldehyde (PFA), Triton X-100, as well as the fluorescent dye phalloidin-FITC were all purchased from Sigma–Aldrich (Milan, Italy). The Vectashield Anti-fade Mounting Medium with 4′,6-diamidino-2-phenylindole DAPI (Vector Laboratories, Peterborough, UK) was used for nuclear staining. All reagents, media supplements, and plastic-ware were supplied as cell-culture tested.

### 2.2. DM and DM/n-HA Nanostructures Synthesis

In brief, DM hybrid nanostructures made of dextran-foils (with a hydrodynamic diameter of 52 ± 4 nm) decorated by γ-Fe_2_O_3_ cores (of 3.0–5.0nm) were prepared by a green-friendly and scalable alkaline co-precipitation method at room temperature, combining a dextran carboxylation and maghemite precipitation reactions modifying a method previously published by Walsh et al. [20]. The superparamagnetic behaviour of the maghemite-decorated dextran hybrid architectures (DM) was recorded at room temperature, showing a non-saturated magnetization (4 emu/g measured at 30 kOe, the maximum value of field applied in the previous study) due to surface anomalies (e.g., defects and spin canting) responsible for a high magnetic frustration. Calcium-deficient hydroxyapatite nanocrystals (Ca/P = 1.5 n-HA) were synthesized in the presence of different amounts of DM hybrid structures (DM/n-HA 1:1, 2:1 and 3:1 weight ratios with respect to the amount of n-HA precipitated in the same reaction performed in the absence of DM). The synthesis was carried out at room temperature and atmospheric pressure as detailed in a previous work [18]. DM and DM/n-HA at different ratios were completely characterized from morphological (TEM), physical–chemical (XRD, ICP, TGA) and magnetic (VSM) point of view as reported in Scialla et al. [18].

### 2.3. Cell Culture and Treatments for Analysis of Cell-Nanocomposites Interaction

Experiments with cells were performed by using the immortalized MG63 cell line (human osteoblast-like cells, ATCC^®^ CRL-1427™) as suitable cell model [19,21]. Cell cultures were maintained in E-MEM medium supplemented with 10% (*v*/*v*) FBS, 2 mM L-glutamine, penicillin (100 U/mL), streptomycin (100 ng/mL), in a water-saturated atmosphere with 5% CO_2_ and 95% air at 37 °C. Once reaching 70–90% confluence (every 2–3 days), cells were washed with D-PBS and detached by adding D-PBS containing 2 mM trypsin-EGTA for 1–5 min, at 37 °C; trypsin activity was stopped by adding an appropriate volume of culture medium (containing α2-antitrypsin). Detached cells were harvested by centrifugation (1200 rpm for 5min, Beckman Coulter Allegra 6R centrifuge with the GH-3.8 swing bucket rotor); the obtained cell pellets were re-suspended in fresh culture medium and redistributed into new flasks. All experiments were performed between passage 3 and 10 of propagation. For the treatments with the nanocomposites, dry DM and DM/n-HA powders were sterilized by ultraviolet (UV) irradiation for 3 h prior to administration to cells. MG63 cells were seeded in multi-well plates, and the day after were incubated for 24, 48 and 72 h with 5 µg/mL of DM powder, or DM/n-HA nanoarchitectures, dissolved in complete growth medium.

### 2.4. Fluorescence Imaging of Cell Cytoskeleton and Nuclei by Phalloidin-FITC/DAPI Double Staining

Cells were seeded on sterile coverslips put at the bottom of 6-well plates; after 24 h (50% ≤ confluence ≤ 70%), cells were treated with nanocomposites according to the described times and concentration. After treatments, cells were fixed by 4% (w/v) PFA in D-PBS at room temperature for 1 h. Fixed samples were permeabilized with 0.5% (w/v) Triton X-100 in D-PBS for 5 min. Then, the following steps were performed: a) wash for 5 min in D-PBS; b) incubation with 1 µg/mL phalloidin-FITC in D-PBS for 20 min (in the dark, under orbital agitation); c) three rapid washes with D-PBS; d) mounting on microscope slides with Vectashield anti-fade mounting medium added with DAPI (for nuclear staining). Fluorescence imaging of cells on the slides was performed by a Zeiss LSM 710 confocal microscopy system, equipped with the ZEN 2009 software (LSM 710 suite).

### 2.5. RNAIsolation and Reverse Transcription

Total RNA was extracted from MG63 cells treated in the absence (control) and in the presence (treated) of DM and DM/n-HA nanocomposites. Briefly, RNA extractions were performed by using the RNeasy Mini Kit (Qiagen, Milano, Italy) according to manufacturer’s instructions, implemented with the on-column Pure Link DNase (Life Technologies, Milano, Italy) treatment. Cells grown on culture dishes were washed twice with D-PBS, and then lysed with the kit lysis buffer by scraping directly on the dish surface. At the end of the extraction protocol, RNA aliquots were kept stored in RNase-free conditions at −80 °C until use. RNA concentrations were calculated by spectrophotometry, and the λ_260_/λ_280_ ratios were determined to evaluate possible protein contamination. Aliquots from the RNA samples were loaded onto agarose gels for checking integrity and any presence of DNA contamination. For cDNA (complementary DNA) synthesis, reverse transcription was performed on 0.25–1 µg total RNA (from each sample) with random primers, using the Bio-Rad iScript Select cDNA Synthesis kit (Bio-Rad) according to manufacturer’s instructions.

### 2.6. Real-Time PCR (qPCR)

For each human gene investigated, the mRNA reference sequences were collected from the NCBI Gene database (https://www.ncbi.nlm.nih.gov/gene/) and were analyzed to identify oligonucleotide tracts to be used as primer pairs in the real-time PCR (qPCR) assays. For each gene, forward and reverse primers were designed in adjacent exons, to avoid possible genomic amplicons. The AmplifX 1.7.0 software(https://inp.univ-amu.fr/en/amplifx-manage-test-and-design-your-primers-for-pcr) was used to validate the selected oligonucleotide sequences that were finally purchased from Eurofins Genomics (Germany). Details of primer sequences are reported in the following Table 1. For qPCR assays, each primer pair was tested for efficiency according to the ‘*amplification efficiency parameters for genes of interest and internal controls*’ proposed by Schmittgen and Livak [22]. qPCR was performed using the iQ SYBR Green Supermix protocol (Bio-Rad, Milan - Italy) with a Rotor-Gene 3000 (Corbett Research, St. Neots, UK) real-time thermal cycler. The 28S ribosomal RNA was used as housekeeping control to normalize mRNA expression. According to Schmittgen and Livak [22], the qPCR output data (i.e., threshold cycle values, Ct) were represented as 2^−ΔCt^ values, i.e., proportional to the amount of the detected target mRNA (ΔCt = target gene Ct–housekeeping Ct). Mean values were calculated from 2 different rounds of qPCR on each of three biological replicates; in graphics, results were reported as mRNA level fold change vs. the untreated control (control fold change = 1).

## 3. Results

### 3.1. Detection of Cytoskeletal Morphology of MG-63 Cells Exposed to DM and DM/n-HA

We have previously assessed the basic cytocompatibility of DM, n-HA and DM/n-HA by evaluating the metabolic activity (viability) of the MG63 human-derived osteoblast-like cells, in the absence of an external magnetic field. In our preceding results, based on MTT assays we detected cytotoxic effects (i.e., reduced cell viability) of DM nanoparticles starting from 5 μg/mL concentration up to 500 μg/mL, after 48 h treatment and up to 72 h post-DM administration [18]. With the same experimental scheme, we assessed the viability of cells in the presence of DM/n-HA nanocomposites synthesized with the 1:1, 2:1, and 3:1 DM/n-HA ratios (in the 1:1 ratio, the amounts of both DM and n-HA were considered to be equal); for all the ratios, the results showed that the cytotoxicity detected for the DM nanoparticles alone was no more observed with the DM-embedding DM/n-HA nanocomposites, hinting a masking effect of DM cytotoxicity by the n-HA component. Moreover, increased cell viability was demonstrated for both the DM/n-HA 2:1 and 3:1 nanocomposites, specifically after 48 h exposure [18].

**Figure 1:**
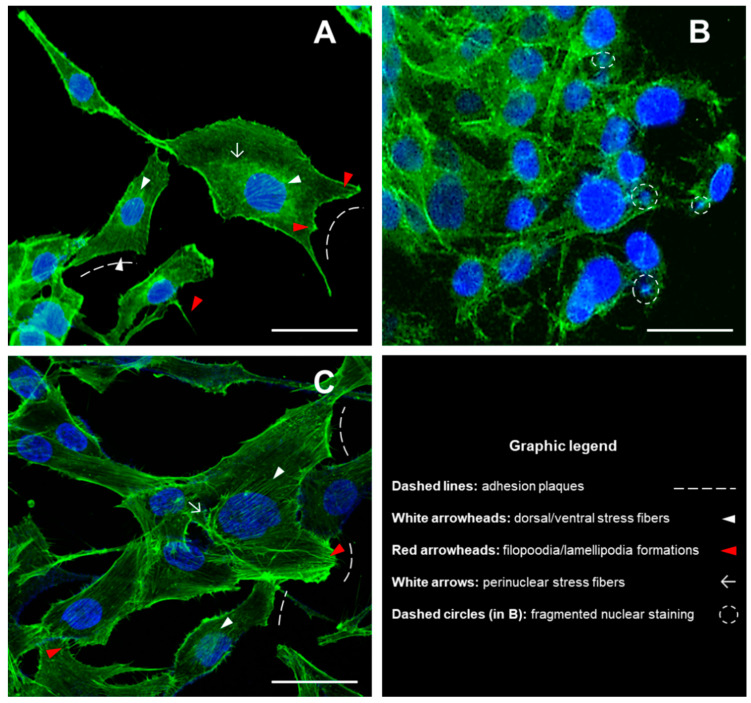
Morphological analysis of cytoskeleton and nuclei in MG63 cells grown for 48 h in the presence of DM nanoparticles and DM/n-HA (2:1) nanocomposites. Fluorescence imaging was performed by using Phalloidin-FITC (green fluorescence) and DAPI (blue) for staining cytoskeleton and nuclei, respectively. The morphology of cytoskeletal structures and nuclear shape has been detected in untreated control cells (**A**), DM-treated cells (**B**) and DM/n-HA-treated cells (**C**). Image capture was performed by a Zeiss LSM 710 confocal microscopy system, equipped with the ZEN 2009 software (LSM 710 suite) [scale bar: 50 µm; magnif. 40X].

Based on this previous evidence, here we show differential responses of cells in the presence of DMs or DM/n-HAs, in terms of morphological features and gene expression variations. Accordingly, we treated cells with 5 µg/mL DM magnetic nanoparticles and DM/n-HA (2:1) for 48 h. After treatments, in untreated control cells the staining of the actin cytoskeleton was well defined according to the regular morphological shape with clear stress fibers and detectable adhesion plaques and cells also displayed normal spherical nuclei (Figure 1A). Contrarily, 48 h after incubation with DM nanoparticles MG63 cells showed tendency to grow in clusters losing their morphological shape, and exhibiting granular appearance of the cytoskeletal staining with increased occurrence of condensed point-like actin structures and, also, no detection of recognizable oriented stress fibers and/or adhesion structures; contrary to the untreated control, the staining of nuclei revealed the occurrence of fragmented formation (Figure 1B). Remarkably, in the presence of DM/n-HA no alterations of the structural morphology could be observed with respect to untreated control cells, after the same 48 h treatment; in fact, cells showed regular structures of the actin cytoskeleton; moreover, the fluorescent staining of cytoskeletal elements such as (ventral, dorsal, perinuclear) stress fibers and adhesion plaques appeared qualitatively thickened and more defined; as reported for the untreated control cells, nuclear staining showed normal nuclei with the absence of any apoptotic onset (Figure 1C).

### 3.2. Effects of DM and DM/n-HA on mRNA Expression of FAK, p53, and DMT1 Genes

By extracting RNA from cells treated for 48 h with the DM nanoparticles and DM/n-HA nanocomposites (1:1, 2:1 and 3:1 ratios), targeted mRNA expression analyses were performed for preliminarily detecting possible (patho-)physiological gene activations. Specifically, variations of the mRNA expression of the *FAK*, *P53* (alias TP53, Tumor Protein 53) and *SLC11A2/DMT1* (SoLute Carrier Family 11, member 2/Divalent Metal Transporter 1) human genes were evaluated by qPCR.

**Figure 2:**
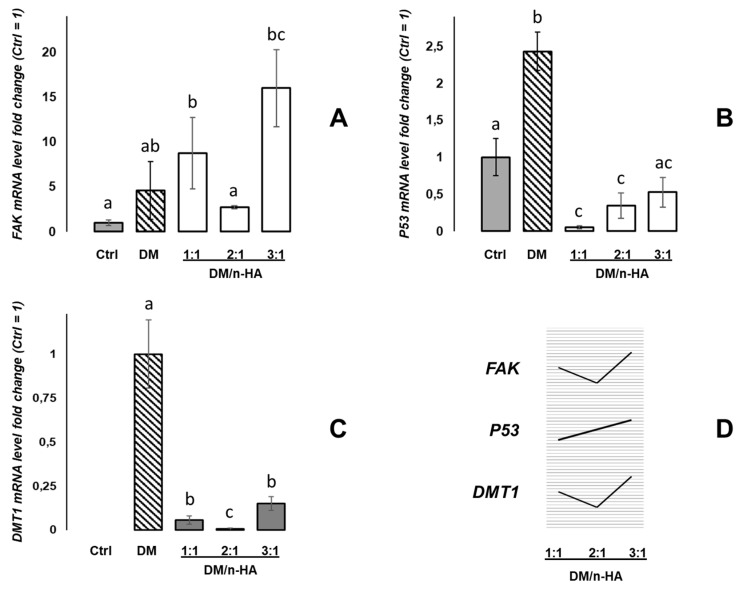
mRNA expression analysis by qPCR in MG63 cells grown in the absence and presence of the 5 µg/mL DM and DM/n-HA (1:1, 2:1, 3:1) for 48 h. The amounts of each target mRNA of the *FAK* (**A**), *P53* (**B**) and *DMT1* (**C**) genes were calculated as 2^−ΔCt^ values obtained from the real-time PCR assays. C_t_ values of the detected mRNA targets were normalized with respect to the 28S RNA housekeeping gene, and were expressed as fold change (*y*-axis) with respect to the control mean values (Ctrl = 1) except for (**C**), where data are expressed as fold change with respect to the DM value (DM = 1). Statistical analysis of variance of the means was assessed by one-way ANOVA and Bonferroni *post-hoc* test [*n* = 4; histograms (mean values) with a common letter are not significantly different with *p* < 0.05 level of significance; histograms (mean values) not sharing any letter are significantly different with *p* < 0.01 level of significance]. In (**D**) graphic summary of the three mRNA expression trends reported for the DM/n-HA 1:1, 2:1, 3:1 experimental conditions.

As reported in Figure 2A, the DM (alone) treatment induced a 4.6-fold up-regulation of the FAK mRNA with respect to the untreated control cells (Ctrl = 1), although without statistical significance. On the other hand, stronger and significant up-regulation was detected for the DM/n-HA 1:1 treatment (+8.8 fold change) but, interestingly, the DM/n-HA 2:1 nanocomposites induced a minor increase of mRNA expression (+2.7), i.e., not statistically different respect to the control; finally, the DM/n-HA at 3:1 ratio showed again a strongly positive variation (+16). Then, the mRNA variations were evaluated for the *P53* gene and relative mRNA expression analyses are summarized in Figure 2B. After treating cells with the DM (alone) nanoarchitectures, a 2.4-fold increase of the P53 transcript was detected. Contrariwise, exposure to the DM/n-HA nanocomposites induced down-regulation of the P53 mRNA levels with respect to untreated cells by all the DM/n-HA ratios, invariably, also statistically significant for the 1:1 and 2:1 ratios.

A third round of expression data was obtained pointing at the detection of effects on DMT1 gene expression as a marker of the iron metabolic pathways, in the MG63 cells exposed to the investigated nanocomposites. As Figure 2C describes, the DMT1 mRNA was not detected in the untreated (control) MG63 cells, which showed not to express constitutively this gene under the adopted experimental conditions. On the contrary, after administration of DM nanoparticles a strong expression of the DMT1 mRNA was detected (fold change = 1 in Figure 2C). Surprisingly, when MG63 cells were exposed to the DM/n-HA nanocomposites, a steep reduction of the DMT1 mRNA levels was identified with respect to the DM-treated cells. In particular, the DMT1 mRNA levels in cells treated with 1:1, 2:1 and 3:1 DM/n-HA underwent −0.94, −0.994 and −0.85 negative fold changes compared to cells exposed to the DM, respectively.

## 4. Discussion

In our previous work, plate-like nanocrystals surface of hydroxyapatite (n-HA) was decorated by dextran-grafted iron oxide nanoarchitectures (DM) in order to provide a magnetic responsiveness to bone-mimicking n-HA. DM hybrid structures were introduced in different weight ratios (DM/n-HA 1:1, 2:1 and 3:1) obtaining nanocomposites able to retain the superparamagnetic behaviour of the DM alone [18]. Consequently, the basic cytocompatibility of the nanocomposites was assessed by evaluating viability and morphology of the human osteoblast-like MG63 cells. The MG63 cell line represents a suitable osteogenic cell model consistently adopted for related research works. Cells of the MG63 line exhibit features of the initial phase of osteogenic differentiation and grow without exhibiting significant contact inhibition; overall, they represent an immature osteoblast phenotype undergoing temporal development in long-term cultures. Moreover, the MG63 cells exhibit insignificant mineralization with respect to other bone-like cell lines; therefore, these cells are particularly useful to study their behavior in the presence of an external source of mineralizing bone elements, such as DM/n-HA [19,21].

We previously reported viability data which hinted a masking effect of the DM cytotoxic potential by the n-HA component. Bearing this in mind, here we investigated the responses of cells in the presence of DMs or DM/n-HAs seeking additional morphological features and gene expression variations supporting the previous observations. In the relevant literature, toxicity of magnetic nanoparticles have been related to changes in cell morphology linked to potential alteration of the cytoskeletal organization [23], although only a few works characterizing nanoparticle-related changes in actin structure have been reported to date. In this view, with respect to the untreated control cells, after treatments with the DM alone we observed: a) high level of disorganization of the cytoskeletal actin fibers (granular formations, occurrence of condensed point-like structures and faint presence of recognizable oriented stress fibers and adhesion structures), b) occurrence of nuclear fragmentation events, and c) clustering growth, all evidence in agreement with the cytotoxic effects of the DM nanostructures [18]. Remarkably, the DM/n-HA nanocomposites (regardless of the 1:1, 2:1 or 3:1 ratios) induced no alterations of the cytoskeletal structures, no occurrence of abnormal/apoptotic nuclei, and no clustering growth, as observed in untreated control cells. This morphological evidence suggests, intriguingly, that the n-HA counterpart might provide a “shielding” component against the DM toxic interactions. Also, the qualitative observations of the cytoskeleton show that a general thickening of stress fibers might occur in MG63 cells after 48 h of incubation with DM/n-HA thus suggesting that enhanced cellular reactivity might be elicited with possible activation of mechano-transduction pathways that transfer inwardly directed signals to cells. Of course, these hypotheses would deserve further quantitative and molecular assessment.

Then, to further understand the differential impact of DM or DM/n-HA nanocomposites in terms of patho-physiological or physiological activations, we performed some specific expression analyses by real-time PCR assays, to identify variations of mRNA levels in MG63 cells undergone 48 h exposure to the different nanoparticles.

First, we analyzed the mRNA expression of *FAK* gene; the FAK activation can be involved in early cell growth phases and intracellular signal transduction pathways triggered in response to ligands or to cell interactions with ECM elements [24]. Although DM did not elicit significant mRNA variations with respect to the untreated control, we found different levels of mRNA variations induced by the three DM/n-HA ratios, with the 1:1 and 3:1 ratios inducing up-regulation of FAK and the 2:1 inducing expression levels comparable with the untreated control. Noteworthy, on one hand diverse patterns of the involvement in mechano-transduction of FAK signaling have been described in MG-63 cells [25,26]; on the other hand, the FAK pathways have been described to be variously activated and influenced by structurally different n-HA-containing composites [27,28]. In this view, remarkably, the 2:1 DM/n-HA type seems to provoke lower levels of activation, resulting more “silent” versus the 1:1 and 3:1 types. Intriguingly, this latter results could be related to the structural differences among the different DM/n-HAs that we previously described in terms of reduced crystallinity associated with the increasing of the DM content [18], implying different cell-material surface interactions which can be investigated as modular parameters to drive signal transduction pathways in cells. Then, we performed the mRNA expression analysis of the *P53* gene (alias *TP53*, Tumor Protein 53) which responds to various cellular stresses by regulating expression of many target genes, and is involved in modulation of processes such as cell cycle arrest, apoptosis, senescence, DNA repair, changes in metabolism; P53 is a transcription factor with a key role in genetic stability and therefore crucially interfering with cancer formation [29]. In our hands, the significant up-regulation of P53 mRNA induced by DM is absent when cells are treated with DM/n-HA nanocomposites. This evidence suggests that the DM-related up-regulation of P53 may potentially be “switched off” by the DM/n-HA complexing. This hypothesis is furtherly enforced since the “switching-off” effect seems to fade with a trend following the increasing of the DM/n-HA ratio (i.e., increasing of the DM relative abundance) (Figure 2D). It must be noticed that these results might gain additional significance when considering that P53 is directly involved in the ferroptosis pathways in cancer [30].

Finally, we analyzed the expression of the *SLC11A2/DMT1* gene (SoLute Carrier Family 11, member 2/Divalent Metal Transporter 1) encoding a key carrier protein involved in iron homeostasis and absorption through cell membranes [31]; altered *DMT1* expression/function is associated with unbalanced responses to iron overload/deficiency [32]. Here, while we detected null expression of DMT1 mRNA in untreated MG63 cells, we clearly identified its expression in cells treated with the iron-containing DM nanoarchitectures which can be reasonably considered to be ligands able to induce the activation of cellular iron-sensing systems. Remarkably, the DM/n-HA nanocomposites induced very low levels of DMT1 mRNA expression, regardless of the DM/n-HA ratio, with respect to DM-induced levels. Once again, such results corroborate, intriguingly, the hypothesis that the DM/n-HA complexes may retain a masking (shielding) effect of almost the whole DM-induced activation, in agreement with previous findings and considerations. Also, it is interesting to note the DM/n-HA 1:1, 2:1, 3:1 trend, indicating the 2:1 ratio as the one inducing the lowest (faint) levels of DMT1 mRNA expression (Figure 2D), thus being the more comparable condition to the null expression in the untreated control. In other words, the observation of mRNA expression levels might hint, interestingly, that the 2:1 complex is the one able to “structurally elude” the possible stimulation of the DMT1-mediated Fe-sensing system, much better than 1:1 and 3:1. It can be hypothesized, intriguingly, that this evidence might be strictly related to different structure and better stability of the 2:1 complex possibly avoiding the release and/or exposure of ferrous components better than 1:1 and 3:1, thus deserving further investigation.

## 5. Conclusions

In this work, we give evidence of differential responsiveness of osteoblast-like cells to different nanocomposites, i.e., dextran-grafted iron oxide (DM) nanoparticles and their hybrid with nano-hydroxyapatite counterparts synthesized in different DM/n-HA ratios which show diverse biomimetic potential. The data hint a shielding, or masking, effect exerted by the n-HA component against the DM cytotoxic onsets, and suggest a modular biomimicry of the synthesized nanocomposites respect to the physiological processes of osteoblast-like cells, in agreement with what has been previously demonstrated in terms of cellular viability. The DM/n-HA nanocomposites here in presented, constitute a promising tool, in which osteoconductivity features of n-HA as bone regenerative-supporting biomaterial, together with magnetic functionalities as imaging reporters and therapeutic effectors are combined in a synergic manner, in an all-in one platform. The possibility of monitoring contrast changes, corresponding to bone tissue morphogenesis, by using magnetic resonance imaging (MRI) is gaining importance as it can provide real-time information about tissue regeneration or repair with a non-invasive techniques and using non-ionizing radiation [33,34,35]. In the specific area of bone defects regeneration, DM/n-HA nanocomposites could be exploited as injectable materials [36,37,38] or fillers in 3D composites substitutes [39,40,41,42]. These materials can be used as fixed stations for spatio-temporal delivery of bioactive stimuli in the site of implantation under the influence of an external magnetic field. Overall, these findings could be the basis for exploiting in deep the high biocompatibility of apatite nanocrystals combined with the magnetic responsiveness of DM, optimizing the combination of structural and functional features to synthesize novel ‘switchable’ nano-tools for bone tissue engineering, for finely acting within physiological ranges.

## Figures and Tables

**Table 1:** Details of primer sequences for the qPCR assays. For each gene, the NCBI accession number of the mRNA reference sequence (Ref Seq mRNA; rDNA for the 28S ribosomal gene) used for primer design is reported. Gene-specific nucleotide sequences are reported as forward (sense) and reverse (antisense) primers on the 5′-3′ direction. For each primer pair, the expected amplicon length is reported (PCR product size) as base pairs (bp). Human genes are listed with the official symbols/aliases.

Human Gene	Ref Seq mRNA acc. n.	5′-3′ Sense	5′-3′ Antisense	PCR Product Size (bp)
**SLC11A2/DMT1**	NM_001174125.1	CACTGTGAACTAAAATCAT	CTCCTCCTCAGGAATGGAGA	235
**P53**	NM_000546.5	CCTCCTCAGCATCTTATCCG	GCACAAACACGCACCTCAAA	250
**FAK**	NM_005607.4	ATTAAATGGATGGCTCCAGA	CTCCCACATACACACACCAA	89
**28S**	M27830.1	GACGAGAGGGCGTGCATTC	TAAAATCCCGCGGACGCAAA	138
